# Risk factors for local lymph node metastasis in hepatic alveolar echinococcosis: a 10-year retrospective cohort study

**DOI:** 10.3389/fonc.2026.1932230

**Published:** 2026-09-24

**Authors:** Yierfan Yilihaer, Yilizhati Aimaitijiang, Ainiwaer Aikebaier, Dong Yan, Tuerganaili Aji

**Affiliations:** 1 Department of Hepatobiliary and Echinococcosis Surgery, Digestive and Vascular Surgery Center, First Affiliated Hospital of Xinjiang Medical University, Urumqi, China; 2 The First Ward of Hepatobiliary and Pancreatic Surgery. Tumor Hospital Affiliated to Xinjiang Medical University, Urumqi, China

**Keywords:** alveolar echinococcosis, echinococcosis, lymph node dissection, lymph node metastasis, radical hepatectomy, risk factors

## Abstract

**Background:**

Hepatic alveolar echinococcosis (HAE) is characterised by infiltrative growth and “cancer-like” biological behaviour. Although radical resection remains the standard treatment, the need for prophylactic lymph node dissection is still debated because preoperative identification of metastasis is difficult. This investigation assesses the risk factors associated with local lymph node metastasis in HAE.

**Methods:**

Clinical data were retrospectively reviewed for 623 patients with HAE admitted to our hospital between January 2012 and January 2022. Based on strict screening criteria, 191 patients who underwent radical hepatectomy were enrolled and divided into a local lymph node metastasis (LNM) group (n=55) and a non-local metastasis group (n=136) based on postoperative pathological findings. Univariate group-comparison tests (independent t-test, Mann-Whitney U test, and Chi-square test) were performed to compare baseline and lesion characteristics between the two groups, followed by multivariable logistic regression analysis.

**Results:**

Univariate group-comparison analyses revealed statistically significant differences between the two groups regarding sex (P = 0.004), age (P = 0.027), ethnicity (P = 0.001), concomitant distant organ metastasis (P<0.001), lesion location (P = 0.001), portal vein invasion (P = 0.016), direct bilirubin (P<0.001), and neutrophil percentage (P = 0.012). Multivariate analysis identified female sex (P = 0.004, OR = 2.941, 95% CI: 1.399–6.182), concomitant distant organ metastasis (P<0.001, OR = 5.522, 95% CI: 2.322–13.133), and bilobar lesion involvement (OR = 4.739, 95% CI: 2.129–10.548, P<0.001) as independent risk factors for local lymph node metastasis in HAE. The multivariable model demonstrated acceptable calibration (Hosmer-Lemeshow test, P = 0.143) and discrimination with an AUC of 0.771 (95% CI: 0.697–0.845).

**Conclusions:**

Female gender, concomitant distant organ metastasis, and lesions involving both hepatic lobes (bilobar involvement) are independent risk factors for local lymph node metastasis in HAE. For patients presenting with these factors, intraoperative prophylactic local lymph node dissection is suggested.

## 1 Introduction

Alveolar echinococcosis (AE) is a severe zoonotic parasitic disease caused by *E chinococcus multilocularis*, primarily endemic to Central Asia and Western China (1). Although not a malignant neoplasm, AE is clinically termed ‘parasitic cancer’ due to its malignant biological behaviours, such as infiltrative growth, destruction of adjacent tissues, and potential for regional lymph node involvement and distant organ metastasis (2). The liver is the primary target organ. With disease progression, HAE lesions can invade intrahepatic blood vessels and bile ducts, causing obstructive jaundice or hepatic failure, and can metastasise via the circulatory or lymphatic systems.

Although regional lymph node involvement has been reported in HAE, systematic research on its predictors and management remains limited (3). Lymphatic dissemination has been proposed as a mechanism of regional lymph node involvement (3). Lymph node metastasis represents extrahepatic involvement, but its prognostic implications require careful interpretation (4).

Current clinical practice faces two dilemmas. First, the preoperative assessment and management of hepatic hilar lymph node involvement remain challenging (5). Second, although WHO-IWGE guidelines provide a framework for the treatment of echinococcosis (6), the indications and extent of regional lymph node dissection in HAE remain incompletely defined (3, 4).

In light of this background, we retrospectively analysed clinical data from 623 patients with HAE admitted to the First Affiliated Hospital of Xinjiang Medical University from January 2012 to January 2022. The overall proportion of patients identified with regional lymph node metastasis (LNM) was 8.8% (55 cases). Based on strict inclusion criteria, 191 patients were enrolled and divided into an LNM group (55 cases) and a non-LNM group (136 cases) for statistical analysis. This study aimed to identify factors associated with regional LNM in HAE and provide risk-stratification information to support diagnosis and surgical decision-making.

## 2 Materials and methods

### 2.1 Patients

A retrospective review was conducted on 623 patients with hepatic alveolar echinococcosis (HAE) admitted to the First Affiliated Hospital of Xinjiang Medical University between January 2012 and January 2022. The study protocol was approved by the Ethics Committee of the First Affiliated Hospital of Xinjiang Medical University (Approval No. 20190226-139) and conducted in accordance with the principles of the Declaration of Helsinki. Because this retrospective study analysed de-identified, anonymised clinical database records under an approved protocol, the requirement for active written informed consent was waived by the Ethics Committee.

The inclusion criteria were: (1) patients with confirmed HAE who underwent radical surgery; and (2) patients with pathologically confirmed local lymph node status. The exclusion criteria were: (1) patients with hepatic cystic echinococcosis (CE) or mixed Echinococcus infections; (2) patients who received palliative treatment only (n = 40); (3) patients with poor cardiopulmonary function or Child-Pugh class C liver function (n = 45); and (4) patients who had no preoperative imaging suggesting lymphadenopathy and did not undergo intraoperative local lymph node dissection or sampling (n = 347). Patients who had received temporary or initial palliative treatments at local clinics before being referred to us—such as percutaneous transhepatic cholangial drainage (PTCD) in order to relieve severe jaundice or portal vein embolisation (PVE) to achieve hypertrophy of the future liver remnant (FLR)—but who then successfully had a radical hepatectomy at our centre were considered eligible and were included.

The patients were divided into two groups according to the definite histopathological results obtained after surgery, these results being about whether or not there was metastasis in the local lymph nodes. The local metastasis group included 55 patients and the non-local metastasis group included 136 patients.

### 2.2 Diagnosis: criteria for diagnosing distant organ metastasis (M1)

Concomitant distant organ metastasis (M1 staging) was identified preoperatively based on chest CT (for lung) or brain/abdominal MRI (for brain or other organs), and was postoperatively confirmed by pathological examination of resected specimens or clinical radiological follow-up.

### 2.3 Surgical procedures

A radical hepatectomy was carried out depending on the position of the lesion and its severity in order to provide individualised treatment, this including standard anatomical resections (such as hemihepatectomy or trisegmentectomy) as well as ex vivo liver resection and autotransplantation. During the operation, the regional lymph nodes along the hepatoduodenal ligament, the portacaval space, and the celiac axis were routinely examined; any nodes that were enlarged or suspected of being involved were subjected to skeletonised dissection and then sent for pathological examination. In order to ensure that there was no tumour left at the surgical margins, the liver resection was combined with an en bloc resection of the adjacent organs that had been invaded, for example the diaphragm, the pancreas, and the kidney. When there was complex involvement of the blood vessels, thrombectomy was performed or the portal vein and inferior vena cava were reconstructed using either autologous or artificial grafts, and biliary reconstruction was accomplished by repairing the bile duct or by carrying out a bilioenteric anastomosis.

### 2.4 Postoperative management and follow-up

After the operation, the patients were subject to continuous monitoring of their vital signs and liver function. The complications were assessed using the Clavien-Dindo classification. When they were discharged, each patient was told to begin long-term oral therapy with albendazole (10 mg/kg/day) as soon as their liver function had normalised, strictly in accordance with the treatment guidelines. Follow-up evaluations were carried out through outpatient appointments or by telephone interviews, and regular surveillance consisted of blood tests, liver function tests, and abdominal imaging (by ultrasonography or CT) to assess recovery and to keep an eye out for any possible recurrence.

### 2.5 Statistical analysis

The statistical analyses were carried out with SPSS version 26.0 (IBM Corp., Armonk, NY, USA) and with R software (version 4.2.2). To compare the clinical characteristics of the local metastasis and non-local metastasis groups (as shown in **Tables 1**–**3**), the continuous variables were first assessed for normality; if the data were found to be normally distributed, they are given as the mean ± standard deviation (SD) and analysed using the independent samples t-test, whereas if the data were not normally distributed, they are presented as the median (interquartile range, IQR) and compared using the Mann-Whitney U test. The categorical variables were given as frequencies and percentages, and the comparisons were made using either the Chi-square test or Fisher’s exact test, depending on the situation.

**Table 1:** Comparison of demographic and baseline characteristics between groups (N = 191).

Patient characteristics	Local metastasis group (n=55)	Non-local metastasis group (n=136)	Statistical value (*χ*2/*t*/*Z*)	P-value
Age (years, Mean ± SD)	35.53 ± 11.49	40.13 ± 13.53	2.226 (t)	0.027
Sex, n (%)			8.147 (*χ*2)	0.004
Male	17 (30.9%)	73 (53.7%)		
Female	38 (69.1%)	63 (46.3%)		
Ethnicity, n (%)			25.419 (*χ*2)	0.001
Tibetan	32 (58.2%)	32 (23.5%)		
Kazakh	10 (18.2%)	38 (27.9%)		
Han	5 (9.1%)	40 (29.4%)		
Hui	2 (3.6%)	4 (2.9%)		
Kyrgyz	3 (5.5%)	5 (3.7%)		
Mongolian	2 (3.6%)	12 (8.8%)		
Uyghur	1 (1.8%)	2 (1.5%)		
Salar	0 (0.0%)	3 (2.2%)		
Residence in endemic area, n (%)			1.028 (*χ*2)	0.311
Endemic area	50 (90.9%)	129 (94.9%)		
Non-endemic area	5 (9.1%)	7 (5.1%)		
Disease duration (months), Median (IQR)	12.00 (1.00–48.00)	4.00 (1.00–24.00)	-1.530 (Z)	0.126

**Table 2:** Comparison of lesion characteristics and invasive features between groups (N = 191).

Patient characteristics	Local metastasis group (n=55)	Non-local metastasis group (n=136)	Statistical value (*χ*2/*t*/*Z*)	P-value
Distant organ metastasis, n (%)			16.115 (*χ*2)	<0.001
With metastasis	19 (34.5%)	14 (10.3%)		
Without metastasis	36 (65.5%)	122 (89.7%)		
Lesion location, n (%)			14.613 (*χ*2)	0.001
Left hepatic lobe	8 (14.5%)	29 (21.3%)		
Right hepatic lobe	25 (45.5%)	87 (64.0%)		
Both left and right lobes	22 (40.0%)	20 (14.7%)		
Solitary or multiple, n (%)			0.848 (*χ*2)	0.357
Solitary	30 (54.5%)	84 (61.8%)		
Multiple	25 (45.5%)	52 (38.2%)		
Portal vein invasion, n (%)			5.792 (*χ*2)	0.016
Yes	36 (65.5%)	111 (81.6%)		
No	19 (34.5%)	25 (18.4%)		
Hepatic vein invasion, n (%)			0.068 (*χ*2)	0.794
Yes	25 (45.5%)	59 (43.4%)		
No	30 (54.5%)	77 (56.6%)		
Hepatic artery invasion, n (%)			0.068 (*χ*2)	0.794
Yes	25 (45.5%)	59 (43.4%)		
No	30 (54.5%)	77 (56.6%)		
Inferior vena cava invasion, n (%)			0.016 (*χ*2)	0.900
Yes	31 (56.4%)	78 (57.4%)		
No	24 (43.6%)	58 (42.6%)		
Biliary tract invasion, n (%)			2.378 (*χ*2)	0.123
Yes	18 (32.7%)	61 (44.9%)		
No	37 (67.3%)	75 (55.1%)		
Long diameter (cm, Mean ± SD)	12.93 ± 4.52	11.48 ± 4.90	-1.906 (t)	0.058
Short diameter [cm, Median (IQR)]	9.20 (7.70–10.93)	8.67 (5.73–11.68)	-1.035 (Z)	0.301

**Table 3:** Comparison of preoperative laboratory findings and nutritional status between groups (N = 191).

Parameters	Local metastasis group (n=55)	Non-local metastasis group (n=136)	Statistical value (*Z*/*χ*2)	P-value
Liver function tests				
Total bilirubin (*μ*mol/L)	15.41 (9.85–37.97)	17.90 (10.13–41.85)	-0.287 (Z)	0.781
Direct bilirubin (*μ*mol/L)	2.17 (0.30–7.16)	5.88 (3.12–20.68)	-4.497 (Z)	<0.001
Indirect bilirubin (*μ*mol/L)	9.95 (7.27–19.88)	10.60 (6.52–21.98)	-0.678 (Z)	0.498
AST (U/L)	36.30 (25.69–65.06)	29.70 (20.50–74.60)	-1.019 (Z)	0.308
ALT (U/L)	38.10 (22.49–74.90)	31.70 (16.70–81.06)	-0.496 (Z)	0.620
Alkaline phosphatase (U/L)	167.19 (102.65–402.60)	164.90 (96.13–359.78)	-0.376 (Z)	0.707
Hematological indices				
Neutrophils (%)	58.70 (53.00–66.90)	63.85 (55.80–72.08)	-2.500 (Z)	0.012
Lymphocytes (%)	25.10 (18.70–30.80)	23.65 (15.63–27.78)	-1.461 (Z)	0.144
NLR	2.34 (1.72–3.58)	2.70 (2.01–4.61)	-2.148 (Z)	0.032
Hemoglobin (g/L)	123.00 (105.00–140.00)	119.00 (106.25–136.75)	-0.587 (Z)	0.557
Platelets (109109/L)	252.00 (215.00–332.00)	239.00 (172.00–313.25)	-1.782 (Z)	0.075
APRI	0.36 (0.24–0.65)	0.31 (0.21–0.78)	-0.654 (Z)	0.513
Nutritional status				
Hypoproteinemia, n (%)	15 (27.3%)	48 (35.3%)	1.139 (*χ*2)	0.286

AST, aspartate aminotransferase; ALT, alanine aminotransferase; NLR, Neutrophil-to-Lymphocyte Ratio; APRI, AST-to-Platelet Ratio Index.

A univariate binary logistic regression was carried out in order to assess the individual association between clinical and laboratory parameters, including composite markers such as the neutrophil-to-lymphocyte ratio (NLR) and the AST-to-platelet ratio index (APRI), and local lymph node metastasis (as shown in **Table 4**); demographic background variables (that is, ethnicity and residence area) were left out of the regression analysis. Those variables which reached statistical significance (with a P value less than 0.05) in the univariate analysis (age, sex, distant metastasis, and lesion location) were incorporated into the multivariable model.

**Table 4:** Univariate and multivariate logistic regression analyses of factors associated with lymph node metastasis.

Variable	Univariate OR (95% CI)	Univariate P-value	Multivariate OR (95% CI)	Multivariate P-value
Age (years)	0.972 (0.948–0.997)	0.029	0.974 (0.947–1.002)	0.073
Sex (Female vs. Male)	2.590 (1.334–5.030)	0.005	2.941 (1.399–6.182)	0.004
Residence (Non-endemic vs. Endemic area)	1.843 (0.559–6.077)	0.315	—	—
Disease duration (months)	1.003 (0.997–1.009)	0.308	—	—
Lesion Location (Bilobar vs. Unilateral)	3.867 (1.885–7.932)	<0.001	4.739 (2.129–10.548)	<0.001
Lesion Number (Solitary vs. Multiple)	0.743 (0.394–1.400)	0.358	—	—
Long diameter (cm)	1.065 (0.997–1.137)	0.061	—	—
Short diameter (cm)	1.040 (0.956–1.132)	0.361	—	—
Distant metastasis (Yes vs. No)	4.599 (2.100–10.074)	<0.001	5.522 (2.322–13.133)	<0.001
Portal Vein Invasion (Yes vs. No)	0.427 (0.211–0.864)	0.018	—	—
Hepatic Vein Invasion (Yes vs. No)	1.088 (0.579–2.042)	0.794	—	—
Hepatic Artery Invasion (Yes vs. No)	1.088 (0.579–2.042)	0.794	—	—
IVC Invasion (Yes vs. No)	0.960 (0.511–1.807)	0.900	—	—
Biliary Invasion (Yes vs. No)	0.598 (0.310–1.154)	0.125	—	—
Liver Function Tests				
Total bilirubin (*μ*mol/L)	0.998 (0.994–1.002)	0.336	—	—
Direct bilirubin (*μ*mol/L)	0.995 (0.988–1.002)	0.152	—	—
Indirect bilirubin (*μ*mol/L)	0.993 (0.983–1.003)	0.183	—	—
AST (U/L)	0.999 (0.996–1.002)	0.371	—	—
ALT (U/L)	0.999 (0.996–1.002)	0.476	—	—
Alkaline phosphatase (U/L)	1.000 (0.999–1.001)	0.821	—	—
Neutrophils (%)	0.975 (0.951–1.000)	0.048	—	—
NLR	0.865 (0.751–0.996)	0.044		
Lymphocytes (%)	1.024 (0.991–1.058)	0.157	—	—
Hemoglobin (g/L)	1.006 (0.992–1.021)	0.398	—	—
Platelets (109109/L)	1.001 (0.998–1.004)	0.426	—	—
APRI	1.082 (0.835–1.402)	0.551		
Nutritional Status				
Hypoproteinemia (Yes vs. No)	0.688 (0.345–1.370)	0.287	—	—

OR, odds ratio; CI, confidence interval; IVC, inferior vena cava; AST, aspartate aminotransferase; ALT, alanine aminotransferase; —, not included in the multivariable analysis.

Model Fit and Validation Statistics:.

Omnibus model test: =42.314 *χ*2 = 42.314, *df* = 4, *P* < 0.001

Nagelkerke pseudo-*R*2 = 0.284

Hosmer-Lemeshow test: =12.186*χ*2 = 12.186, *P* = 0.143

AUC (C-statistic) = 0.771 (95% CI: 0.697–0.845)

The explanatory power, fit, and calibration of the model were evaluated using Nagelkerke’s pseudo-R², the Omnibus test, and the Hosmer-Lemeshow goodness-of-fit test, respectively. Model discrimination was determined by computing the area under the receiver operating characteristic curve (AUC, C-statistic) together with its 95% confidence interval (CI) by means of DeLong’s method. The results were given as adjusted odds ratios (OR) together with their 95% CIs. A two-tailed P value less than 0.05 was regarded as statistically significant. These criteria were applied in order to assess the model’s usefulness for clinical interpretation.

## 3 Results

### 3.1 Patient selection and baseline characteristics

623 patients with confirmed HAE were first screened. Following the patient selection protocol detailed in **Figure 1**, 191 patients were included and divided into a group with pathologically confirmed local metastasis (n = 55) and a group without local metastasis (n = 136). Their baseline characteristics are compared in **Table 1**.

**Figure 1:**
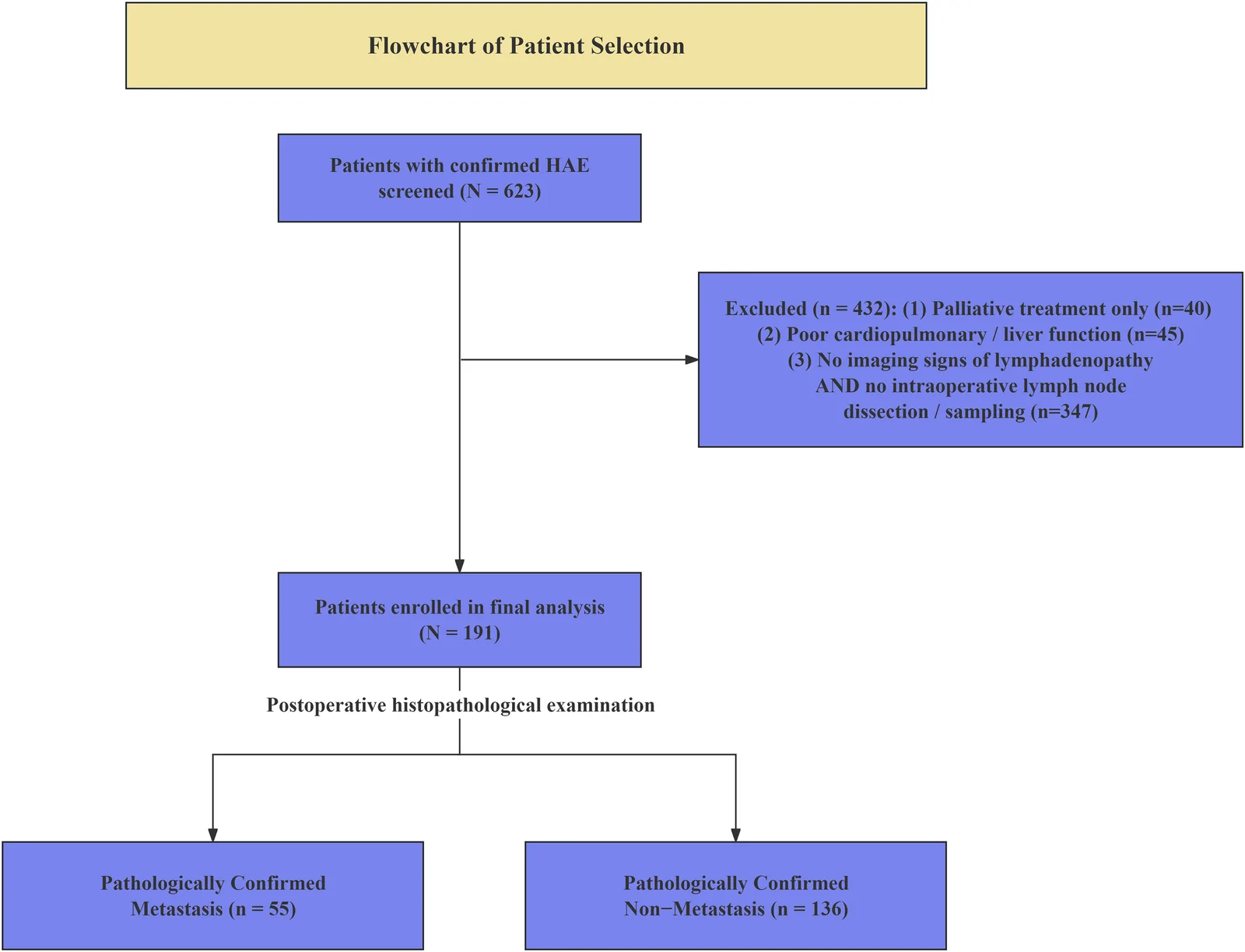
Flowchart of patient selection and histopathological stratification.

As shown in **Table 1**, patients in the local metastasis group were significantly younger (35.53 ± 11.49 vs. 40.13 ± 13.53 years; P = 0.027), had a higher percentage of females (69.1% vs. 46.3%; P = 0.004), and included more Tibetan patients (58.2% vs. 23.5%; P = 0.001) than those in the non-local metastasis group. No significant differences were found in residence in endemic areas (P = 0.311) or illness duration, given as median (IQR) (12.00 [1.00–48.00] vs. 4.00 [1.00–24.00] months; P = 0.126).

In the local metastasis group (n=55), specific clinical features were further examined. Twenty-five patients (45.5%) were asymptomatic at first presentation, while the rest mainly had abdominal pain and distension (n=21, 38.2%) or jaundice (n=7, 12.7%). When preoperative imaging showed lymphadenopathy or suspicious enlarged nodes were found during surgery, regional lymph node dissection or sampling was performed during radical hepatectomy. Fourteen patients (25.5%) had received irregular preoperative albendazole treatment. According to the WHO-PNM classification, all patients in the local metastasis group had intermediate-to-advanced stage disease (P2–P4) with confirmed regional local lymph node metastasis (N1). Distant extrahepatic metastases were found in 19 patients (34.5%), mostly affecting the lungs (n=12, 21.8%), followed by the brain (n=1, 1.8%), pancreas (n=1, 1.8%), and multiple organs (n=5, 9.1%). Preoperative assessments included abdominal ultrasonography and CT scans for all patients, and MRI was performed in 33 cases (60.0%), as detailed in **Table 5**.

**Table 5:** Demographic and clinical characteristics of HAE patients with local lymph node metastasis (n = 55).

Characteristics	No. of patients	Percentage/IQR
Demographics		
Age (years), Mean ± SD	--	35.53 ± 11.49
Sex		
Male	17	30.9%
Female	38	69.1%
Ethnicity		
Tibetan	32	58.2%
Kazakh	10	18.2%
Han	5	9.1%
Other (Kyrghiz, Mongol, Hui, Uyghur)	8	14.5%
Course of illness (months), Median (IQR)	--	12.00 (1.00–48.00)
Clinical presentation		
Symptoms		
Asymptomatic	25	45.5%
Abdominal pain and distension	21	38.2%
Jaundice	7	12.7%
Extrahepatic symptoms	2	3.6%
Medical history		
Previous palliative intervention	19	34.5%
Preoperative irregular albendazole	14	25.5%
Pathological characteristics		
WHO PNM classification		
P2 (P2N1M0 + P2N1M1)	15	27.3%
P3 (P3N1M0 + P3N1M1)	14	25.5%
P4 (P4N1M0 + P4N1M1)	26	47.3%
Distant metastasis (M1)	19	34.5%
Organ involvement (n=19)		
Lung	12	21.8%
Brain	1	1.8%
Pancreas	1	1.8%
Multiple organs	5	9.1%
Comorbidities		
Portal vein cavernous degeneration	1	1.8%
Imaging modality		
USG + CT	55	100.0%
MRI	33	60.0%

### 3.2 Characteristics of lesions

The univariate analysis revealed statistically significant differences between the groups with and without local metastasis regarding distant organ metastasis (P<0.001), lesion location (P = 0.001), and invasion of the portal vein (P = 0.016). Distant organ metastasis was more common in the local metastasis group (34.5%34.5%) than in the non-local metastasis group (10.3%10.3%), and involvement of both hepatic lobes was also more common (40.0%40.0% vs. 14.7%14.7%), as indicated in **Table 2**. The mean long diameter was 12.93 ± 4.52 cm in the local metastasis group and 11.48 ± 4.90 cm in the non-local metastasis group (**Table 2**). The median short diameter was9.20 (IQR: 7.70–10.93) cm in the local metastasis group and 8.67(IQR: 5.73–11.68) cm in the non-local metastasis group (P = 0.301).

### 3.3 Preoperative laboratory findings

The preoperative laboratory results were compared for the two groups. The metastasis group had significantly lower median levels of direct bilirubin (2.17 [IQR: 0.30–7.16] vs. 5.88 [IQR: 3.12–20.68] μmol/L; P<0.001) and a smaller percentage of neutrophils (58.70% [IQR: 53.00%–66.90%] vs. 63.85% [IQR:55.80%–72.08%];P=0.012) than the non-metastasis group. Consequently, the Neutrophil-to-Lymphocyte Ratio (NLR) was significantly lower in the local metastasis group compared to the non-local metastasis group (2.34 [IQR: 1.72–3.58] vs. 2.70 [IQR: 2.01–4.61]; P = 0.032). The AST-to-Platelet Ratio Index (APRI) was comparable between the two groups (0.36 [IQR: 0.24–0.65] vs. 0.31 [IQR: 0.21–0.78]; P = 0.513). No significant differences were detected in the other liver function tests, such as total bilirubin, indirect bilirubin, AST, ALT, and alkaline phosphatase (all P>0.05). The haematological indices, including lymphocyte percentage, preoperative platelet count, and haemoglobin levels, together with the incidence of hypoproteinemia (27.3% [15/55] vs 35.3% [48/136]; P = 0.286), were similar in both groups, as shown in **Table 3**.

### 3.4 Multivariable regression analysis and model validation

The variables that showed statistical significance in the univariate analysis (age, sex, distant metastasis, and lesion location) were incorporated into the multivariable logistic regression model. According to the multivariable binary logistic regression analysis (**Table 4**), female sex, concomitant distant organ metastasis, and involvement of lesions in both lobes were independent risk factors for regional lymph node metastasis in patients with HAE (P<0.05).

Female patients had a higher risk of lymph node metastasis compared to males (OR = 2.941, 95% CI: 1.399–6.182, P = 0.004). Patients with distant metastasis had a 5.522 times higher risk than those without it (95% CI: 2.322–13.133, P<0.001). With regard to lesion location, when unilateral involvement (either the left or right lobe) was taken as the reference point, bilobar involvement was linked with a higher risk of lymph node metastasis (OR = 4.739, 95% CI: 2.129–10.548, P<0.001). The model also took age into account (OR = 0.974, 95% CI: 0.947–1.002, P = 0.073).

In the univariate analysis, NLR was significantly associated with local metastasis (OR = 0.865, 95% CI: 0.751–0.996, P = 0.044), while APRI was not (OR = 1.082, 95% CI: 0.835–1.402, P = 0.551) (**Table 4**). NLR was excluded from the multivariable model to maintain model parsimony and prevent overfitting.

The fit and performance of the multivariable model were assessed. The Omnibus test was statistically significant (χ²=42.314, df=4, P<0.001). The Nagelkerke pseudo-R² was 0.284. The Hosmer-Lemeshow test produced a χ² value of 12.186 and a P-value of 0.143, indicating acceptable model calibration. The forest plot depicting the independent risk factors is given in **Figure 2**, and the clinical risk-prediction nomogram is provided in **Figure 3**. The AUC (C-statistic) of the model was 0.771 (95% CI: 0.697–0.845), as determined by DeLong’s method, as shown in the ROC curve (**Figure 4**). The full results of the univariate and multivariable logistic regression analyses are given in **Table 4**.

**Figure 2:**
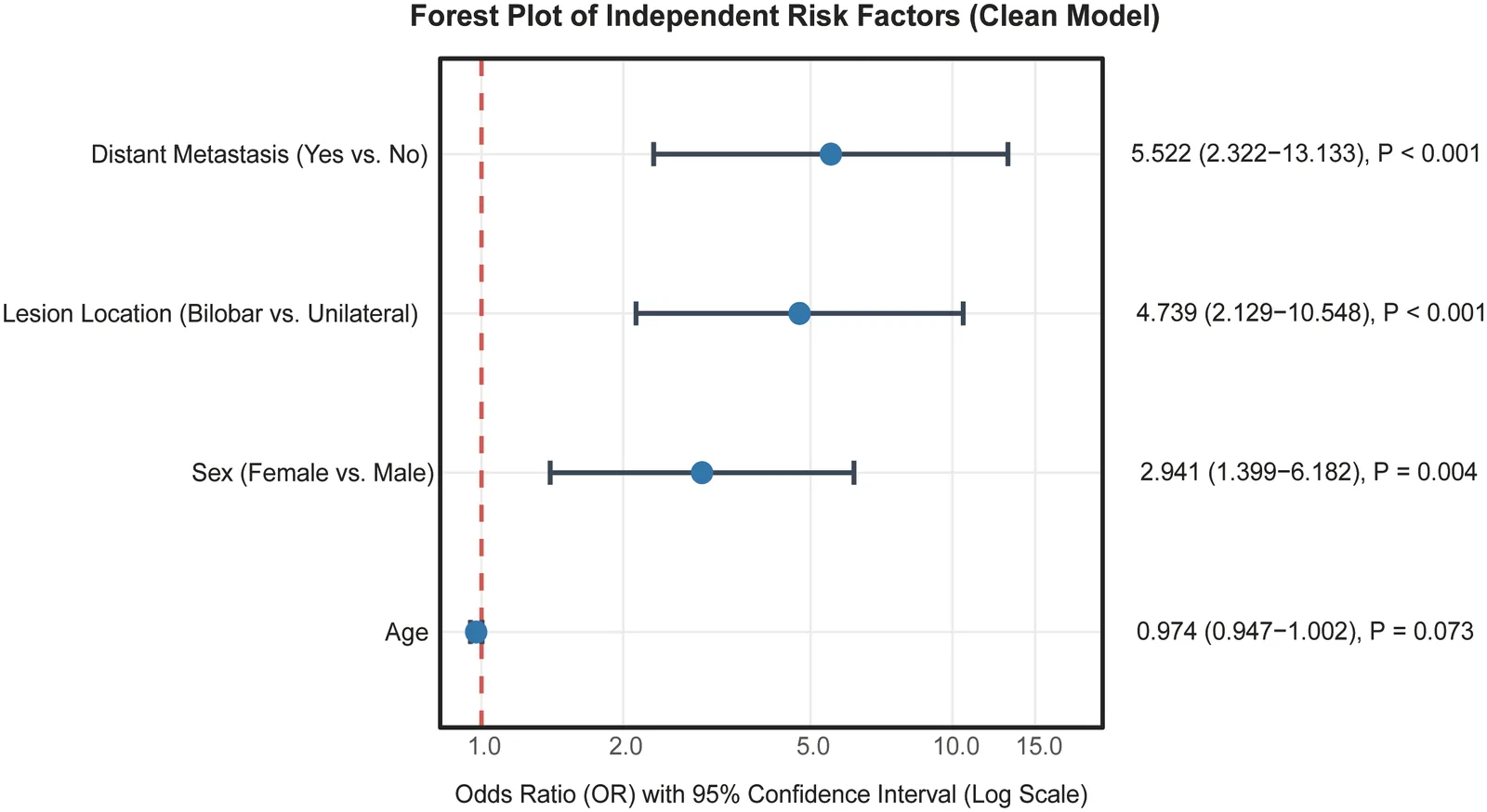
Forest plot of independent risk factors associated with regional lymph node metastasis. The forest plot illustrates the multivariable binary logistic regression results. Female sex (OR = 2.941, 95% CI: 1.399–6.182, *P* = 0.004), concomitant distant metastasis (OR = 5.522, 95% CI: 2.322–13.133, *P* << 0.001), and bilobar lesion involvement (OR = 4.739, 95% CI: 2.129–10.548, *P* < 0.001) were identified as independent risk factors for regional lymph node metastasis. Age (OR = 0.974, 95% CI: 0.947–1.002, *P* = 0.073) was also included in the model. Horizontal bars represent 95% confidence intervals (CIs) on a log scale.

**Figure 3:**
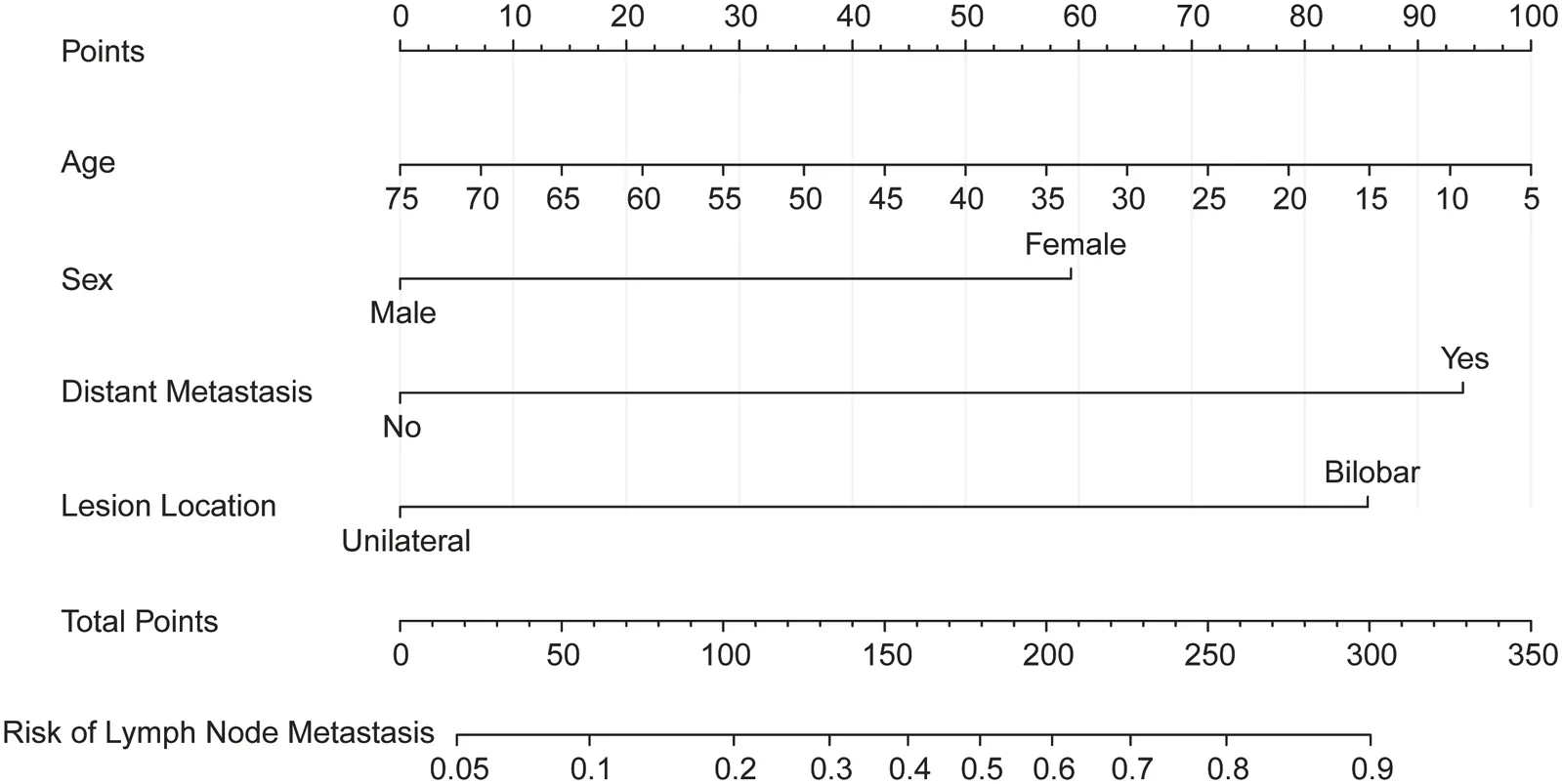
Nomogram for predicting the risk of regional lymph node metastasis in patients with hepatic alveolar echinococcosis. The nomogram was constructed based on the multivariable model, integrating age, sex, local metastasis status, and lesion location. To estimate LNM risk for an individual patient, locate the patient’s value on each variable axis, draw a perpendicular line upward to the “Points” axis to determine the points, sum the points for all variables to obtain the “Total Points”, and draw a perpendicular line downward from the total points axis to determine the estimated probability of lymph node metastasis.

**Figure 4:**
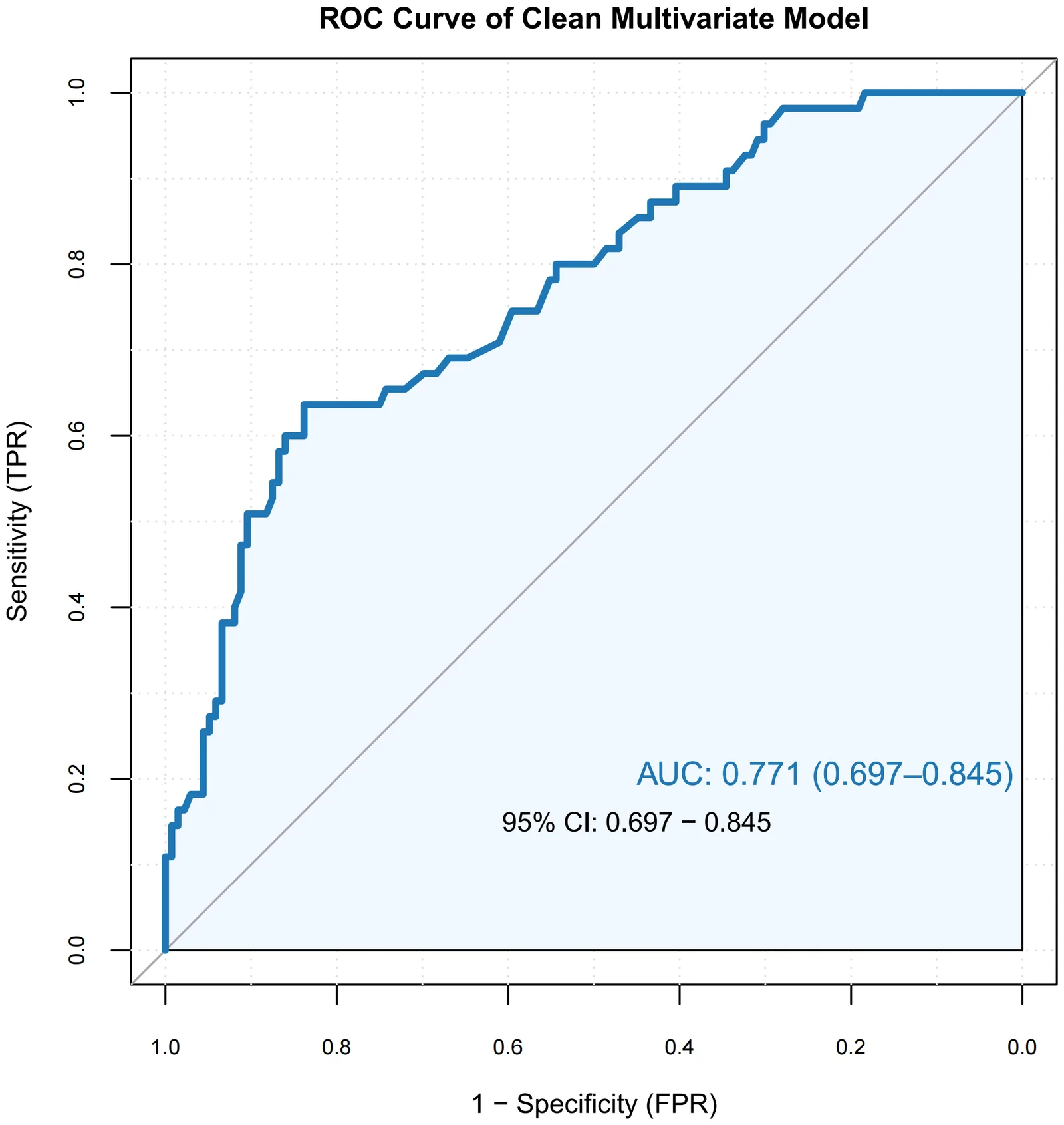
Receiver operating characteristic (ROC) curve of the multivariable predictive model. The multivariable model yielded an area under the ROC curve (AUC/C-statistic) of 0.771 (95% CI: 0.697–0.845) calculated via DeLong’s method, representing acceptable discriminative performance in predicting regional lymph node metastasis in patients with HAE.

## 4 Discussion

AE is a severe zoonotic parasitic disease with infiltrative growth and metastatic potential (1, 2, 7). Following ingestion of E. multilocularis eggs, oncospheres are released in the intestine and reach the liver through the portal circulation (2, 8). Experimental studies have examined the proliferation and dissemination of larval E. multilocularis, including the possible role of germinal cells (9–11). Confirmed or suspected lymph node involvement has also been described in clinical reports (3, 12, 13). This study investigated factors associated with regional LNM in HAE and developed a prediction model to support clinical assessment.

Regarding demographic characteristics, our study highlights a sex-related difference. Our multivariable analysis identified female sex as independently associated with regional LNM (OR = 2.941, 95% CI: 1.399–6.182, P = 0.004). We propose several potential factors that may underlie this association. First, sex-related differences in domestic activities and contact with definitive hosts may influence exposure to infection in endemic regions. However, differences in infection exposure do not necessarily explain the risk of LNM among patients with established HAE. Second, female patients may exhibit distinct immune response patterns related to sex-associated biological factors, potentially including hormonal influences (14). The host immune response to HAE is characterized by a complex balance between protective responses and mechanisms that promote parasite persistence and immune tolerance (2, 15). Sex-related differences in immune regulation may therefore provide a possible explanation for the observed association, although a role for estrogen in HAE-related lymphatic dissemination has not been established. It is possible that female-specific hormonal profiles influence host–parasite interactions, but their contribution to immunotolerance and subsequent lymphatic infiltration remains speculative. Third, increased PD-1 and CTLA-4 expression has been associated with host immune suppression in patients and a mouse model of E. multilocularis infection (16). Whether these pathways interact with sex-related immune differences to influence regional LNM remains unknown. However, direct evidence specifically linking sex hormones to HAE progression remains limited, and this hypothesis warrants further investigation. Intriguingly, HAE patients presenting with regional LNM were significantly younger than their counterparts. Since HAE is characterized by a long clinically silent period (2, 17), infection during childhood is possible in patients diagnosed in their 20s or 30s; however, age at diagnosis alone cannot establish the timing of infection. Age-related differences in host immune responses may contribute to disease progression, but the present study cannot establish that immune immaturity impaired parasite containment or facilitated early lymphatic invasion and dissemination. Additionally, genetic predispositions may contribute to differences in disease progression, although their relationship with younger age at presentation and LNM was not evaluated in this study.

The lesion’s anatomical location was associated with regional LNM. According to multivariable analysis, lesions involving both the left and right lobes (bilobar involvement) were associated with higher odds of regional LNM (OR = 4.739, 95% CI: 2.129–10.548, P< 0.001) compared with unilateral lesions. In HAE, the liver is the primary site of infection (2, 7). Our multivariable analysis revealed that bilobar involvement (lesions spanning both left and right lobes) was associated with regional LNM compared to localized lesions. This finding is consistent with the pathological behaviour of the parasite. AE colonizes the liver via the portal vein and can involve intrahepatic blood vessels, bile ducts, and lymphatic structures through infiltrative growth (2, 9, 10). Hepatic lymph drains in part into the lymph nodes at the hepatic hilum via the deep lymphatic system accompanying the portal structures (18). Consequently, larger lesions, diffuse growth, or bilobar involvement may increase the opportunity for involvement of the hepatic portal vein and micro-lymphatic networks, potentially facilitating lymphatic dissemination. Closely related to this anatomical invasion is a specific vascular alteration observed in our radiological assessments. In cases with significant hilar lymph node involvement, the enlarged lymph nodes can cause external compression and obstruction of the main portal vein. This chronic obstruction promotes the formation of collateral circulation, leading to cavernous transformation of the portal vein (CTPV). As illustrated in **Figure 5**, compression by enlarged regional lymph nodes caused portal venous obstruction and subsequent CTPV in the presented case, highlighting the importance of assessing hilar vascular involvement. HAE complicated by CTPV has also been described in previous reports (19, 20).

**Figure 5:**
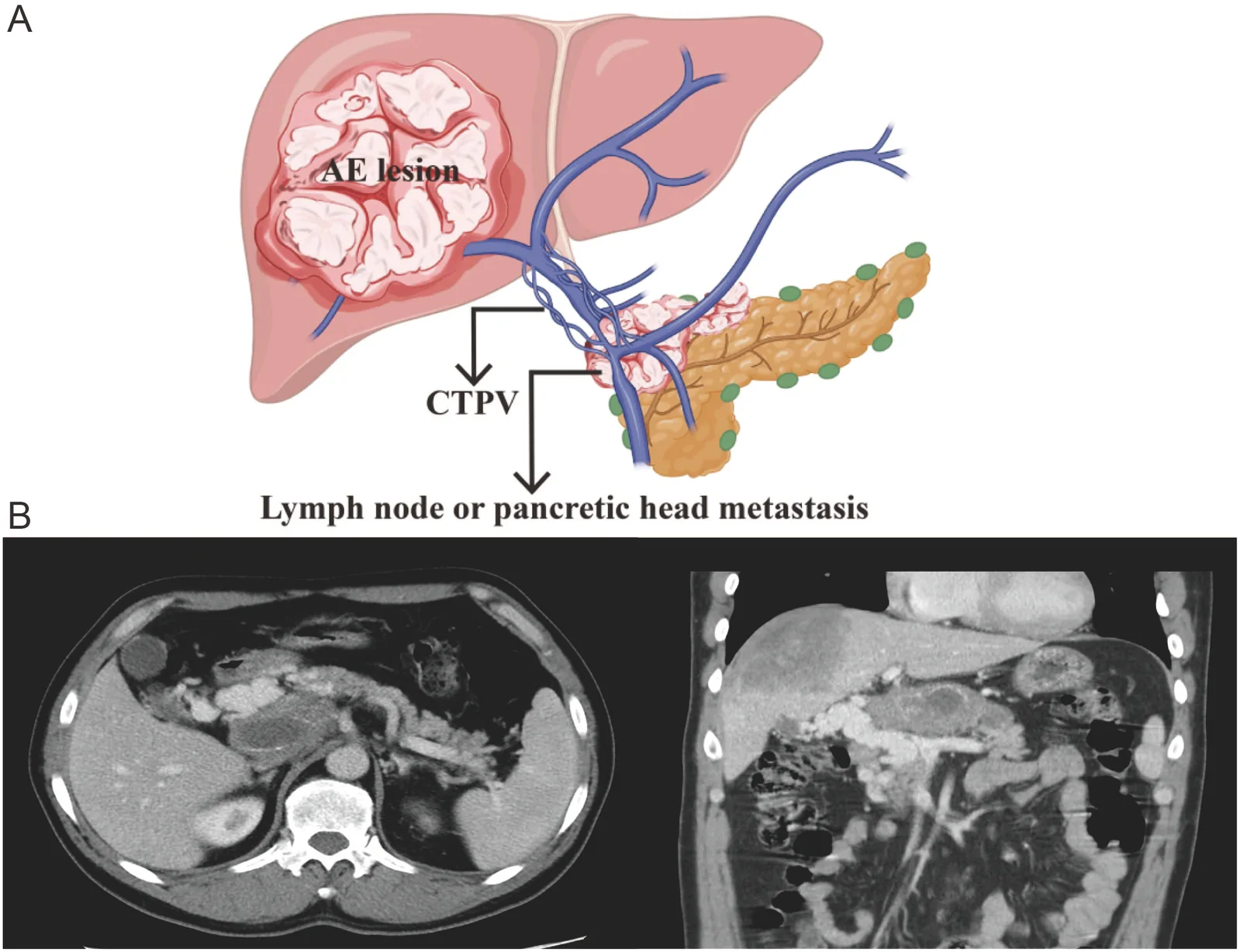
Schematic illustration and contrast-enhanced computed tomography (CT) scans demonstrating cavernous transformation of the portal vein (CTPV) associated with regional metastasis. **(A)** Schematic diagram illustrating the pathological compression of the portal venous system by a primary hepatic alveolar echinococcosis (AE) lesion and its corresponding regional lymph node or pancreatic head metastasis, resulting in portal vein stenosis and subsequent cavernous transformation (CTPV). **(B)** Axial (left) and coronal (right) contrast-enhanced abdominal CT scans showing portal vein obstruction and the development of extensive, tortuous collateral vessels (CTPV) at the porta hepatis and hepatoduodenal ligament.

In terms of geographic spread, HAE is distinct from other parasitic diseases due to its invasive biological characteristics. Our study found that the presence of distant metastases at the same time is an independent risk factor for local metastasis (OR = 5.522, 95% CI: 2.322–13.133, P < 0.001). This link could be bidirectional: on the one hand, distant metastasis always represents advanced disease and involves the vascular system (21–24), thereby damaging the deep lymphatic drainage system; on the other hand, retrograde lymphatic metastasis can take place when the downstream lymphatic vessels are blocked, causing the germinal layer cells to migrate (3). Physical presence of E. multilocularis in lymph nodes is associated with enlarged lymph nodes in alveolar echinococcosis patients (3). Hence, patients with involvement of distant organs (lung or brain) face an increased burden of regional local lymph node metastasis, possibly due to this complex interplay of hematogenous and retrograde lymphatic spread.

In terms of disease dissemination, HAE exhibits invasive biological characteristics (2). Our study found that the presence of concurrent distant metastases was independently associated with regional LNM (OR = 5.522, 95% CI: 2.322–13.133, P < 0.001). This link may reflect more extensive disease: on the one hand, distant metastasis indicates dissemination beyond the primary hepatic lesion and may occur through vascular pathways (2, 8); on the other hand, lymphatic dissemination may contribute to regional lymph node involvement (3). However, whether impaired lymphatic drainage or retrograde lymphatic spread contributes to this association remains uncertain. Physical presence of E. multilocularis in lymph nodes has been described in patients with AE and regional lymph node enlargement (3). Hence, patients with involvement of distant organs (lung or brain) may be more likely to have concurrent regional LNM, possibly reflecting a shared propensity for dissemination rather than a demonstrated bidirectional relationship between distant and lymph node metastases.

To place our findings in a broader clinical context, we compared our results with prior HAE LNM cohorts. The regional lymph node metastasis rate in our screened population was 8.8% (55/623), which was similar to that reported in our previous preliminary descriptive study (Aimaitijiang et al., 2024), in which the reported LNM rate was 8.8% and 69.1% of patients with LNM were female (22). While our previous work was primarily descriptive, the present study advances this research program by developing a multivariable prediction model. Our model yielded an AUC of 0.771. For context, the prediction model recently proposed by Tian et al. (2026) in Frontiers in Oncology achieved a validation AUC of 0.817 (5). However, these AUCs should not be directly compared without accounting for differences in study populations, units of analysis, and validation methods. While Tian et al. focused on individual lymph node imaging features (such as node diameter, nodular calcification, and non-enhancement), our model focuses on macroscopic patient-level predictors (distant metastasis, bilobar lesion location, and female sex), potentially offering complementary information for regional lymph node assessment and individualized surgical planning, rather than establishing an indication for prophylactic lymph node dissection. A CT-based radiomics model for predicting LNM in HAE has also been reported, providing another imaging-based approach to this clinical problem (23).

The clinical relevance of regional lymph node involvement has been examined in studies of lymphadenectomy and long-term surgical outcomes (4, 24). Long-term outcomes after hepatectomy have also been reported in broader AE surgical cohorts (25). Our findings may help identify patients requiring closer regional lymph node assessment, but this study did not evaluate whether prophylactic lymph node dissection improves recurrence or survival. Decisions regarding sampling or removal of suspicious nodes should therefore be individualized according to imaging findings, intraoperative assessment, and surgical feasibility.

Several limitations of this study should be noted. First, this was a retrospective, single-centre study, which may introduce selection and verification bias because patients who had neither preoperative imaging signs of lymphadenopathy nor underwent intraoperative lymph node dissection were excluded, thereby restricting the analysed cohort strictly to pathologically verified patients. Second, due to the relative rarity of HAE with local lymph node metastasis, the sample size of the local metastasis group was relatively small. Third, although the developed risk prediction model demonstrated acceptable performance, we lacked an independent external cohort to validate its clinical utility. Future multi-centre prospective studies are warranted to validate these risk factors.

In conclusion, female gender, the presence of extrahepatic distant metastasis, and bilobar lesion involvement are independent risk factors for local lymph node metastasis in patients with hepatic AE. Clinicians should exercise heightened vigilance in patients presenting with these characteristics. Based on our findings, we suggest that surgeons consider regional lymph node evaluation or dissection in these high-risk patients to potentially improve clinical outcomes.

## Data Availability

The raw data supporting the conclusions of this article will be made available by the authors, without undue reservation.
